# Supplementary material for: Associations between Gross and Fine Motor Skills, Physical Activity, Executive Function, and Academic Achievement: Longitudinal Findings from the UK Millennium Cohort Study
Source: Brain Sci. 2024 Jan 24;14(2):121. doi: 10.3390/brainsci14020121 (PMC10887312; doi:10.3390/brainsci14020121)
Supplement: Supplementary file 1 [file brainsci-14-00121-s001.zip › brainsci-2739232-supplementary.pdf]

Supplementary Materials

Figure S1. Measures of gross and fine motor skills.

Gross Motor Coordination
Infant can sit up without being supported
If infant is put down on the floor, he or she can move about from one place to another
Infant can stand up while holding on to something, such as furniture
Infant can walk a few steps on his or her own
**Fine Motor Coordination**
Infant grabs objects using the whole hand
Infant passes a toy back and forth from one hand to another
Infant can pick up an object using forefinger and thumb only
Infant puts his or her hands together

Table S1. Regression table for decision making quality.

	Model 1	Model 2	Model 3	Model 4	Model 5	Model 6
Socioeconomic status						
Family income	0.08(0.00)***	0.08(0.00)***	0.08(0.00)***	0.06(0.00)**	0.06(0.00)**	0.06(0.00)**
Maternal education	0.08(0.00)***	0.08(0.00)***	0.83(0.00)***	0.06(0.00)**	0.06(0.00)**	0.06(0.00)**
Early motor coordination						
Gross motor coordination		-0.02(0.00)	-0.02(0.00)	-0.02(0.00)	-0.02(0.00)	-0.02(0.00)
Fine motor coordination			0.00(0.00)	-0.01(0.00)	-0.01(0.00)	-0.01(0.00)
Verbal & nonverbal ability						
Naming vocabulary				0.04(0.00)	0.04(0.00)	0.04(0.00)
Picture similarity				0.03(0.00)	0.03(0.00)	0.03(0.00)
Pattern construction				0.11(0.00)***	0.11(0.00)***	0.11(0.00)***
Physical activity						
MVPA					-0.04(0.00)**	-0.04(0.00)**
Self-reported PA					0.06(0.00)**	0.00(0.00)
Constant	(0.01)**	(0.03)***	(0.05)***	(0.05)***	(0.05)**	(0.05)***

Note. SE = Std. Error; **p < .01; ***p < .001; β = Standardized coefficients. Model 1: Family income & maternal education. Model 2: Model 1 + Gross motor coordination. Model 3: Model 2 + Fine motor coordination. Model 4: Model 3 + BAS naming vocabulary & picture similarity & pattern construction. Model 5: Model 4 + MVPA. Model 6: Model 5 + Self-reported PA

Table S2. Regression table for spatial working memory total errors.

	Model 1	Model 2	Model 3	Model 4	Model 5	Model 6
Socioeconomic status						
Family income	-0.14(0.00)***	-0.15(0.00)***	-0.15(0.00)***	-0.11(0.00)***	-0.11(0.00)***	-0.11(0.00)***
Maternal education	-0.11(0.19)***	-0.11(0.19)***	-0.11(0.19)***	-0.08(0.18)***	-0.08(0.18)***	-0.08(0.18)***
Early motor coordination						
Gross motor coordination		-0.02(0.27)	-0.01(0.28)	0.00(0.27)	0.00(0.27)	0.00(0.27)
Fine motor coordination			-0.02(0.39)	-0.00(0.38)	-0.00(0.38)	-0.00(0.38)
Verbal & nonverbal ability						
Naming vocabulary				0.00(.10)	0.00(0.10)	0.00(0.10)
Picture similarity				-0.07(.10)***	-0.07(0.10)***	-0.07(0.10)***
Pattern construction				-0.23(.04)***	-0.23(0.04)***	-0.23(0.04)***
Physical activity						
MVPA					0.01(0.01)	0.01(0.01)
Self-reported PA						0.00(0.34)
Constant	(0.84)**	(2.73)***	(4.91)***	(4.93)***	(5.00)**	(5.15)***

Note. SE = Std. Error; **p < .01; ***p < .001; β = Standardized coefficients.
Model 1: Family income & maternal education. Model 2: Model 1 + Gross motor coordination. Model 3: Model 2 + Fine motor coordination. Model 4: Model 3 + BAS naming vocabulary & picture similarity & pattern construction. Model 5: Model 4 + MVPA. Model 6: Model 5 + Self-reported PA
